# Characterization of Binary Biofilms of *Listeria monocytogenes* and *Lactobacillus* and Their Response to Chlorine Treatment

**DOI:** 10.3389/fmicb.2021.638933

**Published:** 2021-07-14

**Authors:** Magdalena A. Olszewska, Francisco Diez-Gonzalez

**Affiliations:** ^1^Center for Food Safety, College of Agricultural and Environmental Sciences, University of Georgia, Griffin, GA, United States; ^2^Department of Industrial and Food Microbiology, Faculty of Food Science, University of Warmia and Mazury in Olsztyn, Olsztyn, Poland

**Keywords:** *Listeria monocytogenes*, *Lactobacillus*, biofilm structure, microbial interaction, hypochlorite treatment, matrix composition

## Abstract

In nature, *Listeria* may interact competitively and cooperatively with other organisms, resulting in unique spatial organization and functions for cells within the community. This study was undertaken to characterize the biofilm architecture of binary biofilms of *Listeria monocytogenes* and *Lactobacillus* species and to assess their effect on the survival of *Listeria* during exposure to hypochlorite. Three *L. monocytogenes* strains, ATCC 19115 (Lm5), ATCC 19117 (Lm7), and Coleslaw (LmC), were selected and combined individually with three *Lactobacillus* strains: *L. fermentum* (Lf), *L. bavaricus* (Lb), and *L. plantarum* (Lp). In binary Lm-Lp biofilms, the Lm cell counts were similar to single-species biofilms (8.5 log CFU/well), and the Lp cell numbers declined by 1.0 log CFU/well. In the presence of Lb, the Lm cell counts were reduced by 1.5 log CFU/well (*p* < 0.05), whereas the Lf cell counts increased at least by 3.5 log CFU/well. Confocal laser scanning microscopy (CLSM) determined that interspecies interactions significantly affected the spatial organization of three binary biofilms. Biofilm surface-to-volume ratio increased from 0.8 μm^2^/μm^3^ for Lm5 in the monoculture to 2.1 μm^2^/μm^3^ for Lm5-Lp in the dual-species model (*p* < 0.05), and was characterized by a thicker structure with a largely increased surface area. Biofilm roughness increased from 0.2 for Lm7 to 1.0 for Lm7-Lb biofilms (*p* < 0.05), which appeared as interspecific segregation. Biofilm thickness increased from 34.2 μm for LmC to 46.3 μm for LmC–Lf (*p* < 0.05), which produced flat and compact structures that covered the entire surface available. The biomass of the extracellular matrix was higher in the case of some binary biofilms (*p* < 0.05); however, this effect was dependent upon the species pair. When treated with hypochlorite, Lm5 in binary biofilms had an approximately 1.5 log CFU/well greater survival than individually. The unique spatial organization and greater protein production may explain the protective effect of Lp after hypochlorite exposure.

## Introduction

In nature, microbes exist predominantly as communities of sessile cells known as biofilms (Donlan, 2002). Biofilms can be defined as aggregated microbial communities surrounded by a matrix of self-produced extracellular polymeric substances (EPS), which form on a wide variety of surfaces (O’Toole et al., 2000; Kim and Lee, 2016). In these distinctly structured and organized communities, cells coordinate their behavior and are capable of demonstrating specific functions (Rutherford and Bassler, 2012). Biofilms can play a positive role and are beneficial commercially for the immobilization technology, e.g., for the removal of crude oil from wastewaters (Nie et al., 2016). In contrast, biofilms can also have detrimental effects due to their strong antimicrobial tolerance and contribution to the persistence of pathogenic microorganisms in the food processing environment, thus, biofilms of *Escherichia coli* O157:H7 (e.g., Zhao et al., 2013), *Salmonella* spp. (e.g., Yang et al., 2016), *Pseudomonas aeruginosa* (Kim et al., 2008), *Bacillus cereus* (Ryu and Beuchat, 2005), and *Listeria monocytogenes* (e.g., Guilbaud et al., 2015) have attracted special attention over the years.

Cells originating from the biofilms formed in different locations of a food processing facility represent a potential source of food contamination, and *L. monocytogenes* is a bacterium of the greatest concern because of the high morbidity and mortality rate of foodborne listeriosis. Despite the thorough cleaning and disinfection applied, *Listeria* is repeatedly found at such a facility. Various studies have reported that *L. monocytogenes* can be present in food processing areas for very long periods (Saá Ibusquiza et al., 2012; Nowak et al., 2017; Rodríguez-Campos et al., 2019) and these persistent isolates may produce more biofilm than transient ones (Rieu et al., 2008; Kostaki et al., 2012). In addition, *L. monocytogenes* is able to form biofilms on different surfaces (Zhao et al., 2013; Alonso et al., 2014), representing a serious concern for the food industry.

The presence of other bacterial species along with a pathogenic bacterium may increase biofilm formation to the benefit of the pathogen by providing protection. However, different interactions, including synergistic and antagonistic, have been reported for *L. monocytogenes* biofilms with *Pseudomonas putida* (Saá Ibusquiza et al., 2012), *Salmonella enterica* (Kostaki et al., 2012), *Staphylococcus aureus* (Rieu et al., 2008), and *Vibrio parahaemolyticus* (Chen et al., 2019). Certainly, there is a correlation between the interspecific interactions and spatial organization of microorganisms in multi-species biofilms. According to the latest literature, a strong interdependence favors intermixing or layered structure, whereas weak interdependence is reflected in interspecific segregation and layered structure with patchy patterning, and finally, mutual inhibition resulted in a decreased biomass with patchy patterning or interspecific segregation (Liu et al., 2016). Moreover, functional properties like antimicrobial tolerance may be associated with the spatial architecture of biofilms. The limitation of agent penetration reflects the importance of the matrix shape and the three-dimensional organization of cells in protecting biofilm inhabitants (Kim et al., 2008). Evidence of spatial organization of mixed-species biofilms carrying *L. monocytogenes* and how and if the pathogen can be privileged in surviving environmental challenges like disinfection measures is unfortunately very limited, hence, further studies with various bacterial species are needed.

Obtaining knowledge on biofilms composed of multiple bacterial species can be accomplished by confocal laser scanning microscopy (CLSM) as it offers the direct *in situ* and non-damaging investigation of native multicellular structures (Bridier et al., 2010). CLSM proved to be suitable for the characterization of the single-species biofilms of *L. monocytogenes* and opportunistic pathogens such as *E. coli*, *P. aeruginosa*, *S. aureus*, and *Enterococcus faecalis* (Bridier et al., 2010). In addition to the biofilm architecture of *L. monocytogenes*, this study aimed to correlate the genetic lineages of the isolates with structural diversity of their biofilms (Guilbaud et al., 2015). Given the importance of the co-existing microbiota for *L. monocytogenes* establishment and survival, we were particularly interested in the characterization of biofilms harboring *L. monocytogenes* in the presence of commensal or spoilage-associated genera such as *Lactobacillus*. Lactobacilli often share the same niche with *Listeria*, i.e., soil, plant material, and the food-processing environment including meat and dairy-related industrial environments where non-starter lactobacilli are introduced. Resident lactobacilli were shown to either protect or inhibit *L. monocytogenes* in the biofilms (Van der Veen and Abee, 2011; Pérez-Ibarreche et al., 2016). This ambiguous behavior prompted us to further investigate the characteristics of the binary biofilms of *L. monocytogenes* (Lm) with *Lactobacillus* spp., all selected on the basis of their biofilm formation capacity, and to assess the effect of cohabitation on the survival of *Listeria* during exposure to hypochlorite. Our approach specifically aimed at describing the spatial parameters to provide more knowledge on the architecture of Lm-carrying biofilms and if and how Lm can survive chemical disinfection depending on the *Lactobacillus* species present in the system. *Lactobacillus* species, such as *L. fermentum*, *L. bavaricus*, *L. sakei*, or *L. plantarum*, used here for biofilm formation screening represent the non-starter and resident lactobacilli found in the food-related industrial environments. The variability between the biomass (cell counts), structural parameters, and matrix production of binary biofilms was shown and comprehensively discussed as to if particular characteristics are involved in the hypochlorite tolerance of Lm.

## Results

### Screening Biofilm Formation

**FIGURE 1:**
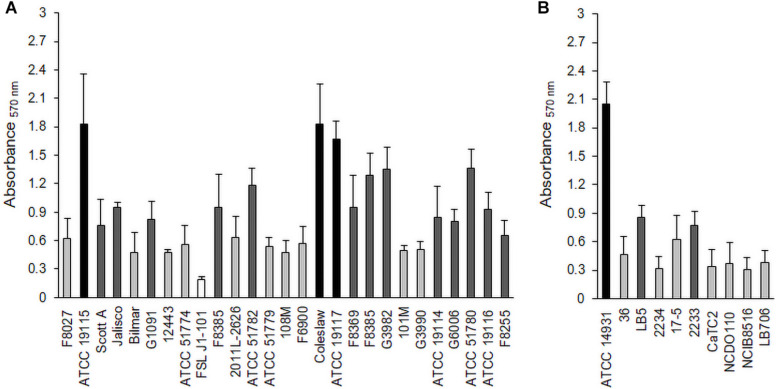
Biofilm formation by *L. monocytogenes* strains **(A)** and *Lactobacillus* species **(B)** under aerobic conditions. Biofilms were quantified by the crystal violet (CV) assay at 570 nm. Black bars represent strong biofilm producers; dark gray, moderate biofilm producers; light gray, weak biofilm producers; and white, non-adherent.

*L. monocytogenes* strains (Table 1) and *Lactobacillus* species (Table 2) were tested for their biofilm formation abilities, and results indicated that both bacteria were better able to form biofilms aerobically than anaerobically (Supplementary Tables 1, 2). Average OD readings of *L. monocytogenes* biofilms grown aerobically and stained with crystal violet were very different among strains (Figure 1A), which suggested diverse biofilm-forming capabilities. These results allowed us to select three biofilm producers, i.e., *L. monocytogenes* ATCC 19115 (Lm5), *L. monocytogenes* ATCC 19117 (Lm7), and *L. monocytogenes* Coleslaw (LmC), for biofilm architecture and matrix localization studies. Also, three were selected among the *Lactobacillus* species, *L. fermentum* ATCC 14931 (Lf), *L. bavaricus* LB5 (Lb), and *L. plantarum* CaTC2 (Lp) (Figure 1B), and used as secondary species for *Listeria* cells in binary biofilms.

**TABLE 1:** The 27 *Listeria monocytogenes* strains used in this study.

**Strain**	**Isolate origin (if known)**	**Serotype (if known)**	**References**
F8027	Food (celery)	4b	UGA
19115	Human	4b	ATCC
ScottA	Food (raw milk)	4b	UGA
Jalisco	Food (cheese)		UGA
Bilmar	Food (hot dog)		UGA
G1091	Coleslaw outbreak	4b	UGA
12443		1/2a	UGA
51774	Human	1/2a	ATCC
FSLJ1-101		1/2a	UGA
F8385		1/2a	UGA
2011L-2626	Cantaloupe outbreak		CDC
51782	Food (dairy)	3a	ATCC
51779	Food (dairy)	1/2c	ATCC
108M	Food (meat)		UGA
F6900	Clinical isolate, deli meat		UGA
Coleslaw	Food (Coleslaw)	4b	UGA
19117	Animal (sheep)	4d	ATCC
F8369	Food (corn)	1/2a	UGA
F8385	Food (carrots)	1/2b	UGA
G3982	Human, outbreak linked to Mexican-style cheese	4b	UGA
101M	Food (beef)		UGA
G3990	Human, outbreak linked to hot dog	4b	UGA
19114	Animal	4a	ATCC
G6006	Human, outbreak, IL, United States	1/2b	UGA
51780	Food (cheese)	1/2b	ATCC
19116	Animal (chicken)	4c	ATCC
F8255	Food (peach/plum)	1/2b	UGA

*UGA, University of Georgia; ATCC, American Type Culture Collection; CDC, Centers for Disease Control and Prevention.*

**TABLE 2:** The *Lactobacillus* spp. strains used in this study.

**Species**	**Strain**	**Other code**	**Isolate origin (if known)**	**References**
*L. fermentum*	14931	B1 28		ATCC
	36	B-9338		UGA
*L. bavaricus*		LB5		UGA
*L. plantarum*	2234			Silliker
	17-5	LB3 8014		UGA
	CaTC2	LB7	Animal-derived foodstuff	USDA ARS
*L. coprophilus*	2233			Silliker
*L. buchneri*	NCDO110	B-1837	Food (tomato pulp)	USDA ARS
*L. malefermentans*	NCIB8516	B-1861	Food (fermented beverages)	USDA ARS
*L. sakei*		LB706		UGA

*ATCC, American Type Culture Collection; UGA: University of Georgia; USDA ARS, Agricultural Research Service United States Department of Agriculture Silliker-Silliker Laboratories.*

### Biofilm Growth Observation in Monoculture Biofilms

**FIGURE 2:**
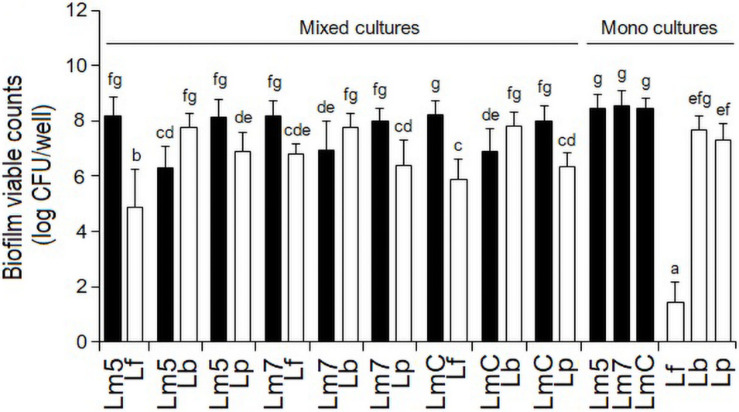
Biofilm viable counts of *L. monocytogenes* strains and *Lactobacillus* species in mono and binary culture conditions grown in brain heart infusion supplemented with 0.005% manganese sulfate. Biofilm cells were quantified by enumeration following detachment, re-suspension, and agar plating. Different letters indicate a significant difference at a *p*-value of 0.05. *L. monocytogenes* strains ATCC 19115 (Lm5), ATCC 19117 (Lm7), and Coleslaw (LmC); *Lactobacillus fermentum* ATCC 14931 (Lf), *L. bavaricus* LB5 (Lb), and *L. plantarum* CaTC2 (Lp).

As monocultures, there was no difference in the biofilm cell counts among the Lm strains, reaching levels of 8.4 log CFU/well (Lm5) and 8.5 log CFU/well (Lm7, LmC) in BHI (*p* > 0.05) (Figure 2). Lb and Lp formed their biofilms at the levels of 7.7 and 7.3 log CFU/well, respectively, whereas Lf failed to develop biofilms (∼2.0 log CFU/well) despite its abundant planktonic growth in BHI (data not shown) and biofilm formation in MRS (∼8.0 log CFU/well).

### Biofilm Growth Observation in Binary Biofilms

However, when Lf was grown in combination with Lm strains, its final cell counts increased by 3.5 log CFU/well. Still, all the binary Lm–Lf biofilms had from 1.4 to 3.3 log CFU/well more *Listeria* than Lf (*p* < 0.05) (Figure 2). Likewise, binary biofilms with Lp also resulted in a greater contribution of *Listeria* (from 1.2 to 1.6 log CFU/well; *p* < 0.05). However, Lp analyses did not show significant differences in the cell counts between its single-species derivative and in combination with Lm5 (*p* > 0.05). In contrast, Lb had a higher contribution to the mixed-species biofilm than any *Listeria* (0.8–1.5 log CFU/well; *p* < 0.05), and its cell count was slightly higher than in the single-species counterpart.

### Biofilm Resistance to Chlorine Treatment

**FIGURE 3:**
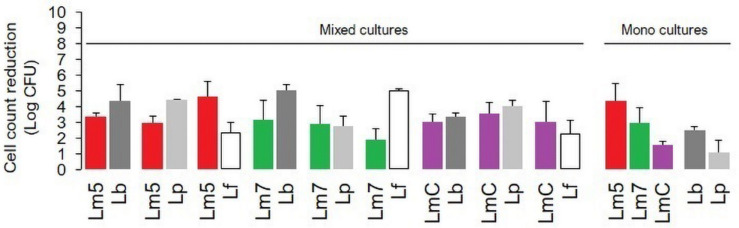
The impact of chlorine (50 ppm) on the viability of *L. monocytogenes* strains (Lm5/Lm7/LmC) and their combinations with *Lactobacillus* species (Lb/Lp/Lf) in mono and binary culture biofilms assessed with the plate counting method and exhibited as log cell reductions. Lf is not represented as a monoculture (data not obtained). Results were means of at least three independent experiments conducted on different days. Abbreviations: see Figure 2.

**FIGURE 4:**
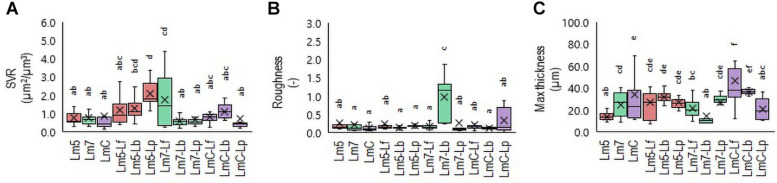
Box plots of the biofilm structural parameters: surface-to-volume ratio (SVR) **(A)**, roughness average **(B)**, and maximum thickness **(C)** obtained for *L. monocytogenes* strains and their combinations with *Lactobacillus* species after confocal data processing of Syto^®^ 9-labeled biofilms. The boxes range from the 25th to the 75th percentile and are intersected by the median line. Whiskers extend below and above the box range, from the lowest to the highest values, respectively. Averages are indicated by a cross symbol (×). Different letters indicate a significant difference at a *p*-value of 0.05. Abbreviations: see Figure 2.

The inactivation of mono and binary biofilms by chlorine is shown in Figure 3. Three Lm strains (Lm5/Lm7/LmC) individually and in combination with Lb/Lp/Lf were tested. A concentration of 50 ppm chlorine reduced the count of Lm5 and Lm7 by 4.4 and 2.9 log CFU, respectively, in the monocultures, but no more than 3.3 log CFU were killed in the binary biofilms. In dual-species models, Lm5 increased its survival by 1.5 and 1.0 log when co-cultured with Lp and Lb, respectively. Lp was the most resistant to chlorine, resulting in a reduction of 1.1 log CFU, however, it declined greatly (4.4 log CFU) in combination with Lm5. No such protective effect was observed for Lm7, with a reduction of approx. 3.0 log CFU. However, once in combination with Lf, the reduction of Lm7 decreased to 1.9 log CFU and the inactivation of Lf was comparatively high. Note that this pair scored first in terms of Lf cell contribution (Figure 2) and second in SVR increase (Figure 4). LmC itself yielded the lowest reduction (1.5 log CFU), and no increase in survival was observed when in the binary biofilms but with equal reduction levels between 3.0 and 3.5 log CFU instead.

### 3-D Biofilm Architecture

To determine which Lm biofilms were associated with which structural parameters, a Principal Component Analysis (PCA) was performed. The PCA revealed a separation of Lm biofilms along with their loadings in PC 1 and PC 2 (Supplementary Table 3). Given that the specific patterning of bacteria may lead to an increased biomass and enhanced tolerance toward antimicrobials compared to their component species individually, three binary biofilms were the most noteworthy. Lm5 when grown in combination with Lp yielded the closest proximity to the surface-to-volume ratio (SVR), whereas Lm7 when grown with Lb approached roughness. In contrast, LmC with Lf was strongly attributed to maximum thickness. Accordingly, differences were observed between these biofilms and those formed individually. The SVR increased from 0.8 μm^2^/μm^3^ for Lm5 in monoculture to 2.1 μm^2^/μm^3^ in the binary culture biofilm (*p* < 0.05) (Figure 4A). For the monoculture biofilm, the roughness coefficient was 0.2 for Lm7, whereas in the binary biofilm, the roughness was 1.0 (*p* < 0.05) (Figure 4B). Considering the thickness for LmC biofilms, values of 34.2 and 46.3 μm in the mono and binary cultures, respectively, were obtained (*p* < 0.05) (Figure 4C). However, we also found that in the case of Lm5, the thickness increased from 13.8 to 26.6 μm in its binary biofilm with Lp (*p* < 0.05). This was not the case for Lm7 in the binary biofilm with Lb, but rather a contrary tendency was observed.

**FIGURE 5:**
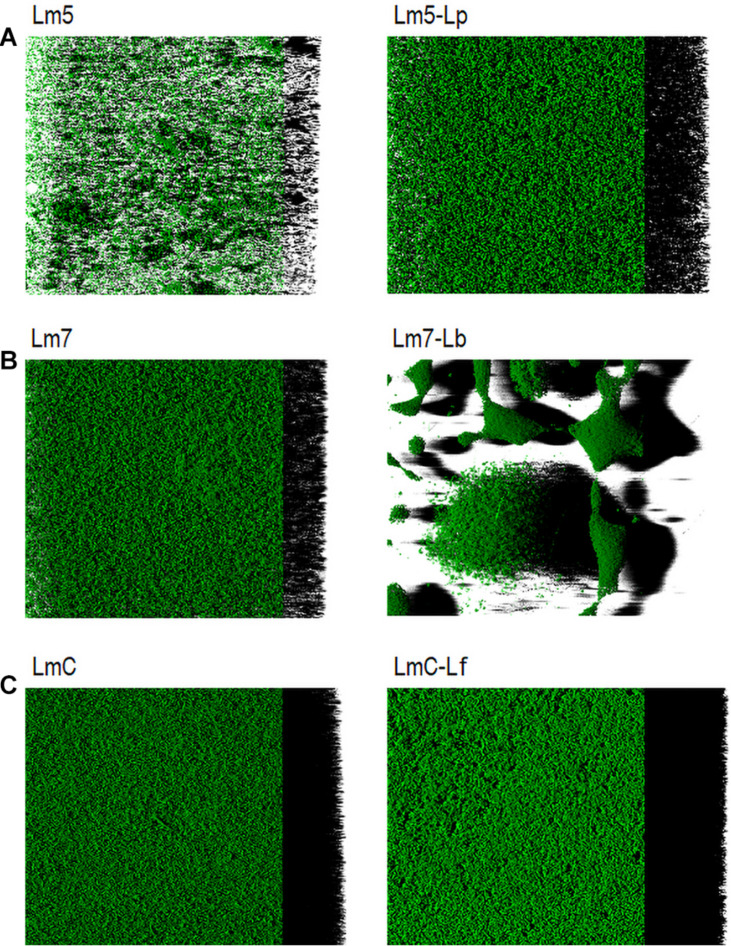
Three-dimensional biofilm structures of *L. monocytogenes* strains in mono and binary culture conditions with different *Lactobacillus* species obtained from confocal *z*-stacks using the ZEN 2.3 software. **(A)** Lm5 individually and in combination with Lp. **(B)** Lm7 individually and in combination with Lb. **(C)** LmC individually and in combination with Lf. These images present the shadow projection on the right. The biofilms were labeled with Syto^®^ 9, a cell permeant green fluorescent nucleic acid marker. Abbreviations: see Figure 2.

Representative biofilm structures for three Lm strains and their binary combinations (Lm5-Lp, Lm7-Lb, and LmC-Lf) are presented in Figure 5. The images correspond to the 3-D reconstructions acquired from confocal stacks, with shadow projections on the right. In the monoculture, LmC colonized the entire substratum and its biofilm was the thickest, whereas Lm5 formed small scattered cell clusters and overall thin biofilms. Lm5 with Lp formed denser structures, closely associated with one another. Lm7 and Lb grew independently and often formed separate microcolonies, despite the ability of Lm7 to cover the entire surface as in the monoculture. LmC with Lf formed flat and thick structures. The biofilms of *Lactobacillus* species had variable structures (Supplementary Figure 1). Lp developed thin biofilms that covered almost the entire surface, whereas the biofilm image of Lb showed an uneven coverage since the cells tended to create clumps. Lf produced rough structures, but only in the MRS broth. For review, their structural parameters are also available in Supplementary Figure 2.

### 3-D Biofilm Matrix Localization

**FIGURE 6:**
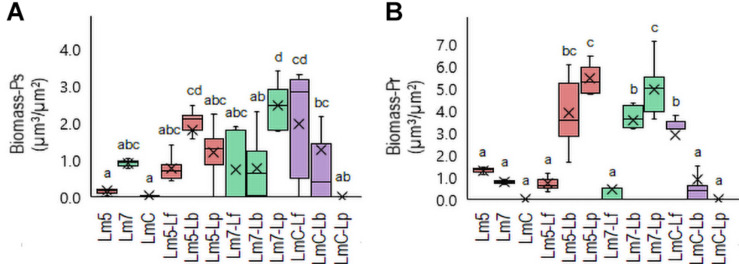
Box plots of the biomass: polysaccharides (Ps-**A**) and proteins (Pr-**B**) obtained for *L. monocytogenes* strains and their combinations with *Lactobacillus* species after confocal data processing of FITC-WGA and SYPRO^®^ Ruby-labeled biofilms. Box and whisker description: Figure 3. Different letters indicate a significant difference at a *p*-value of 0.05. Abbreviations: see Figure 2.

**FIGURE 7:**
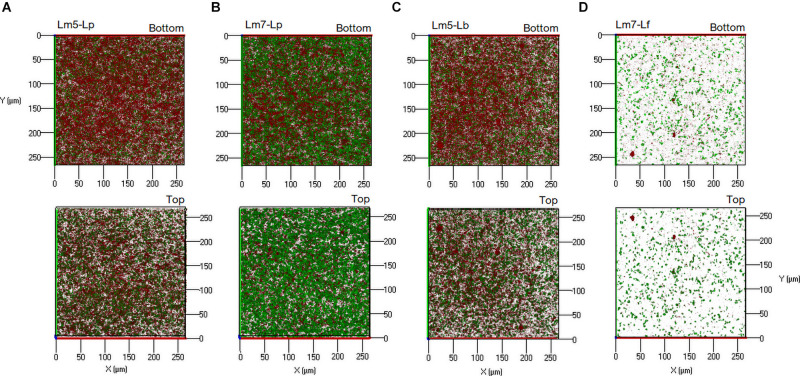
Three-dimensional projections of double stained biofilms: FITC-WGA (green, for exopolysaccharide detection) and SYPRO^®^ Ruby (red, for protein detection). **(A)** Lm5 in a binary biofilm with Lp. **(B)** Lm7 in a binary biofilm again with Lp. **(C)** Lm5 in a binary biofilm with Lb. **(D)** Lm7 in a binary biofilm with Lf. Abbreviations: see Figure 2.

New staining revealed the 3-D biofilms based on the non-cellular biomass components polysaccharide and protein (Figures 6, 7). Some binary biofilm pictures displayed a substantial boost in the matrix production since they were variables with closer proximity to biomass variables (Supplementary Table 4). In particular, Lm5 and Lm7 in the binary biofilms with the same secondary species, Lp, stood out from the other biofilms (Figure 6). Individually, Lm5 produced more proteins and Lm7 more polysaccharides. Then, polysaccharide biomass increased from 0.2 to 1.2 μm^3^/μm^2^ and from 0.9 to 2.5 μm^3^/μm^2^ (Figure 6A), and protein biomass from 1.3 to 5.4 μm^3^/μm^2^ and from 0.8 to 4.9 μm^3^/μm^2^ in Lm5-Lp and Lm7-Lp biofilms, respectively (*p* < 0.05) (Figure 6B). LmC itself was not capable of producing the matrix under the conditions tested, nor the stimulation by Lp was observed. However, more matrices were observed when Lf was present, since values of 1.9 and 3.5 μm^3^/μm^2^ were obtained for polysaccharides and proteins, respectively (*p* < 0.05). Visual inspection revealed the presence of highly fluorescent red-stained aggregates, thus, more protein than polysaccharide fraction for Lm5-Lp (Figure 7A) and brightly green-labeled components and, thus, an opposite relationship for Lm7-Lp (Figure 7B). Another best matrix producer was again Lm5, this time with Lb, because an increase to 1.8 and 3.9 μm^3^/μm^2^ (polysaccharide and protein) was observed and the visual inspection also showed that red-stained aggregates prevailed (Figure 7C). In contrast, the biomass of protein and polysaccharide obtained for the Lm7-Lf pair was comparatively small, with no indication of a stimulating effect (Figure 6).

## Discussion

The use of high throughput methods which allow the detailed characterization of the native architecture of biofilms is crucial to contribute to the understanding of microbial interactions and advancement in the field of biofilm research (Azeredo et al., 2017). Previous studies have discussed the significance of biofilm formation by *L. monocytogenes* (Borucki et al., 2003; Pilchová et al., 2014; Renier et al., 2014; Heredia and García, 2018) and 3-D structures have been previously described, i.e., a monolayer of cells, unstructured multilayers, a knitted-chain network, or honeycomb-like structures (Bridier et al., 2010; Renier et al., 2014; Guilbaud et al., 2015). Here, we applied a high throughput microscopic method for a high-resolution imaging of the biofilms of *L. monocytogenes* strains in mono and binary culture conditions with *Lactobacillus* species. We were especially interested in deciphering biofilms based on the different structural and functional parameters. They were important to better understand the complex behavioral and survival strategies of *L. monocytogenes* biofilms.

In this work, *L. monocytogenes* formed biofilms containing several small aggregates and the emergence of cell clusters with a high degree of substratum coverage as single strain biofilms. The architecture of mixed communities was diverse in particular within three combinations, as emphasized by the SVR, roughness, and thickness parameters (Figure 4). As reflected in the increased thickness and SVR, cells of Lm5 and Lp arranged together indicates that when biofilms became thicker, the surface area of the biofilm actually increased (Figure 5A). Note that cooperation or exploitation results in an increased biomass, while competition leads to a decreased biomass in the mixed-species compared to single-species biofilms; the Lm5-Lp pair corresponded to the former. Although Lm5 was dominant in the biofilm, this particular combination had the lowest difference in the cell counts between the two species and the highest overall cell contribution than any other binary community. Considering the structural parameters, the surface-to-volume ratio was closely associated with the Lm5-Lp pair (Figure 4A).

The surface-to-volume ratio reflects what fraction of the biofilm is exposed to the nutrient flow, hence indicating how the biofilm adapts to the environment (Heydorn et al., 2000). Also, single cells and small cell aggregates naturally have a higher surface-to-volume ratio than larger microcolonies, suggesting that Lm5-Lp tended to mix together to a certain degree and form packed clusters decorated with loosely attached cells. Lm5-Lp pair also had strong matrix-producing abilities, in particular, toward proteins. Interesting observations on the interactions could be seen between Lm strains and Lf, where lactobacilli were markedly stimulated in the presence of *Listeria*. Depending on the Lm strain, these communities revealed differences in their 3-D architectures, suggesting the most compact structure with LmC.

Certainly, microbial interactions among different species have a significant impact on the growth potential, survival, individual behavior of each species, and the biofilm structure-function relationships (Kostaki et al., 2012). In our study, Lm tend to cooperate, compete, and communicate with Lp, Lb, and Lf in the binary biofilms. Interactions are cooperative, when interspecies interactions lead to benefits for one or all interacting species and are crucial for the overall biofilm fitness. Cooperative interactions include the secretion of enzymes or metabolic cross-feeding, and often lead to the specific spatial organization of different species in biofilms (Tan et al., 2017). Hence, this scenario matched the Lm-Lp pair than any other pair. Competition results in a decreased productivity for all or some interacting species. They compete for space and nutrients in an indirect strategy where fast growers deplete resources from slow competitors or in a direct fight with competing species, for example, by secretion of antimicrobials (Yang et al., 2011). This was the most likely scenario when Lb was present in the system.

To further continue, Lb competed with Lm strains and cells of Lm7 and Lb most probably attached as separate microcolonies in a spatial organization known as interspecific segregation (Figure 5B). Their structure generally presented the greatest roughness and the smallest bacterial thickness, thus, biomass resulting in a spatial structure with initial species segregation driven by exploitative or competitive interactions. Because *Listeria* residing in a binary community greatly suffered from the interaction with Lb, this might also fall into interference competition, hence, further studies need to be carried out to identify the compounds promoting this phenomenon. Such spatial organization where Lm forms its own microcolonies has been found in biofilms with the Gram-negative bacterium, *Comamonas testosteroni* (Carpentier and Chassaing, 2004). Similarly, competitive advantage was demonstrated for *V. parahaemolyticus* when with Lm cells because its favorable location on the surface layers resulted in overgrowing the partner strain (Chen et al., 2019). Likewise, in the presence of the antagonist produced by *B. cereus*, the number of Lm cells was lower, contributing to competitive interactions (Alonso et al., 2020). However, the number of cells was again higher in the presence of non-antagonist producers, suggesting a cooperative behavior between species. Lm was also dominant in the presence of *S. aureus*, showing an intimate association and an increase in the number of cells in the cell-free supernatant (Rieu et al., 2008).

In addition, signaling molecules-based communication known as quorum sensing (QS) plays an important role in intra- and interspecies interactions. Since biofilms comprise high concentrations of cells, QS is considered a crucial form of interaction leading to specific physiological activities for the cells (Rutherford and Bassler, 2012). Cells secrete autoinducers (AIs), which accumulate in the environment as the population density increases and further govern the gene expression like *luxS*. It has been demonstrated that *luxS* in *Lactobacillus acidophilus* is upregulated in response to viable cells and cell-free supernatant of Lm (Moslehi-Jenabian et al., 2011). This triggered important effects on the behavior and functionality of co-existing bacteria. It could be hypothesized that a higher transcription of the gene might affect the adherence of lactobacilli to the substratum positively and competitive exclusion might affect the adherence of Lm. This could largely explain the observations made about Lm-carrying biofilms when Lf was present in the system.

Antimicrobial tolerance resulting from different interspecific interactions and the spatial organization of cells in multi-species biofilms has not been fully elucidated. In our study, a link has been found between microbial interactions and matrix production capacity by cells. This positive effect was the greatest in Lm-carrying biofilms when co-cultured with Lp, with only one exception, i.e., much production did not occur for LmC. This signifies the important stimulating role of a secondary species in a strict strain-dependent manner as well as an essential role of the matrix in providing a favorable habitat for bacteria to co-exist and protect against antimicrobial action.

In our disinfection experiment, we used chlorine due to its broad-spectrum bactericidal activity, which is recommended to be used at a concentration of at least 50 ppm to achieve a safe and effective sanitizing effect. Limited reports have been published on the hypochlorite resistance of Lm in a mixed-species biofilm, nor comparison with results of a single species biofilm, making it difficult to judge upon the protection of secondary species over Lm. Previous work on disinfectant resistance of Lm-carrying biofilms showed that it obtained a higher resistance to benzalkonium chloride (BAC) when co-cultured with *P. putida* (Saá Ibusquiza et al., 2012) or *L. plantarum* (Van der Veen and Abee, 2011). In the latter study, both species were more resistant to the disinfection treatments whereas in our study, it was just one of them.

We observed that Lm5 was more resistant to hypochlorite than its single species counterpart while Lp was largely diminished. The Lm5-Lp pair was characterized by a thicker structure with an increased surface area and increased matrix production, in particular, the protein fraction. We could hypothesize that the close association with its partner species, Lp, provided protection toward Lm as well as the elevated protein production created a diffusion barrier to hypochlorite penetration. In addition to physical barrier, the antimicrobial can be inactivated due to chemical interaction with proteins, thus reducing its effect on the underlying cells. The former scenario makes more sense for the Lm 7-Lf pair where Lf reached the greatest cell counts while in co-culture with Lm7, and this pair was also characterized by a high surface-to-volume ratio.

An intimate association between Lm7 and Lf comparable to that of Lm5-Lp indicates that the increased resistance of Lm in the binary biofilm is closely related to the 3-D organization of cells and here, refers to the localization of partner species. Peripheral species may be more susceptible to antimicrobial treatments, and no benefit is provided for the killed species, i.e., Lf. The fact that Lf was significantly increased when with Lm and reduced after the treatment suggests the shielding effect of Lf toward privileged Lm in the biofilm.

Another species that provided protection for Lm5 was Lb and this association resulted in an abundant protein production. Given that Lm5 was overgrown by Lb like any other Lm strain, a key role here may be played by still the favorable localization of Lm and the reaction-diffusion limitation of the protein matrix. Of note, Lm7 when co-cultured with Lb, which revealed confluent growth areas where bacteria formed clumps separated from each other, was not more tolerant to hypochlorite. This indicates that Lb directly fights Lm7 either by secreting anti-listerial compounds or creating an environment suitable for the emergence of antimicrobial susceptible cells. It again proves that an intimate association leading to a particular organization of cells with a greater surface area confers the privilege to one of the species, Lm. This way, the generation of structural data can enable a clearer understanding of biofilm traits.

## Conclusion

*L. monocytogenes* exhibits co-dependence with other bacteria that may help avoid the removal or inactivation by disinfectants in food processing environments. We argued that the cohabitation with *Lactobacillus* spp. may result in an increased survival against hypochlorite. Most importantly, the intimate association related to the certain spatial organization of cells and matrix production rich in protein fraction may explain the protective effect of *Lactobacillus* over *Listeria*. Thus, this study contributes to a better understanding of the behavior of this pathogen and it may help to inform better strategies for the use of antimicrobial treatments against *Listeria* biofilms. Obtained results also highlight that to improve disinfection, it is crucial to further characterize those bacterial associations that occur in nature. Yet, a special focus on the spatial organization of cells within multi-species biofilms may help reveal the molecular mechanisms underlying the interspecies interactions.

## Materials and Methods

### Bacterial Strains, Media, and Preparation of Inoculum

Bacterial strains used in this study are listed in Tables 1, 2. They include 27 *L. monocytogenes* strains and 10 *Lactobacillus* spp. isolated from different origins and stored in the collection of the Center for Food Safety, University of Georgia, United States. Before use, *Listeria* strains were resuscitated from −80°C stocks in a tryptic soy broth (TSB; Difco Laboratories, Sparks, MD, United States) and incubated for 24 h at 34°C. Lactobacilli strains were transferred to De Man, Rogosa, and Sharpe broth (MRS; Acumedia, Lansing, MI) and incubated for 24 h anaerobically at 30°C. Anaerobic conditions were obtained by incubating the cultures inside sealed jars containing anaerobic atmosphere packs (Mitsubishi^TM^ AnaeroPack, Thermo Fisher, Waltham, MA). Working cultures were prepared by adding 100 μL of each pre-culture to 10 mL of TSB or MRS broths and incubating at 34°C or 30°C for 18 h and, finally, diluting it down in 0.1% buffered peptone water (BPW; Difco) (ca. 10^6^ CFU mL^–1^), in order to be used as the inoculum for the biofilm development assays. For the binary culture conditions, the inocula of *L. monocytogenes* and *Lactobacillus* spp. (1:1) were mixed so that a cocktail was used.

### Screening Biofilm Formation

To select the best biofilm producers, the biofilm formation of 37 strains altogether was evaluated with a crystal violet assay. Different oxygen requirements for *Listeria* and lactobacilli were used when biofilms were grown under aerobic and anaerobic incubation conditions. Aliquots (200 μL) of each bacterial suspension (in BPW) were transferred into wells of 96-well polystyrene (PS) microtiter plates (Costar, Corning, NY) and plates were incubated for 3 h at 34°C, under static conditions. Subsequently, the liquid cultures were removed with a pipette and each well was washed twice with BPW to remove unattached cells. Volumes of 200 μL of the TSB or MRS were added, and plates were incubated in an aerobic atmosphere and the added vent or inside of sealed jars containing anaerobic atmosphere packs (Mitsubishi^TM^ AnaeroPack, Thermo Fisher, Waltham, MA) for 24 h at 34°C under static conditions. Liquid cultures were once again removed and each well was washed with BPW. For biofilm fixation, 200 μL of ethanol (≥99.5%, v/v) were loaded. After 15 min, ethanol was pipetted and plates were air-dried. Subsequently, 200 μl of 1% (wt/vol) crystal violet solution (Sigma-Aldrich, St. Louis, MO, United States) were added to each well, and plates were incubated for 15 min at room temperature. After washing with water, the plates were dried and the wells were loaded with 200 μL of acetic acid 33% (v/v) (Merck) to release and dissolve the stain. Absorbance was read using a Cytation^TM^ 3 imaging reader (BioTek Instruments, Inc. Winooski, VT, United States) at 570 nm. The experiment included up to four replicate wells and was repeated three times. Optical density (ODs) readings were measured and compared with the cut-off OD (ODc), which was described as three standard deviations above the mean OD of the negative controls containing only media. For the interpretation, the following classifications were used: no biofilm producers (OD ≤ ODc), weak biofilm producers (ODc < OD ≤ 2 × ODc), moderate biofilm producers (2 × ODc < OD ≤ 4 × ODc), and strong biofilm producers (4 × ODc < OD) (Stepanović et al., 2000).

### Biofilm Growth and Chlorine Treatment

The bacterial growth of the best biofilm producers of *L. monocytogenes* strains (3) and *Lactobacillus* species (3) on PS microtiter plates under mono and binary culture conditions was evaluated by using the drop plate (DP) method. All six strains were selected for assessing the chlorine effect. The biofilms on PS microtiter plates were first grown as described in the previous section. We used brain heart infusion (BHI; Neogen, Lansing, MI, United States) broth for the co-cultures and supplemented it with 0.005% manganese sulfate (MnSO_4_; Merck) to meet the high Mn requirements of *L. plantarum* for growth (Van der Veen and Abee, 2011). After growth, liquid cultures were removed and wells were washed with BPW to obtain mature biofilms. Wells receiving in-well treatments were added with 200 μl of sodium hypochlorite solution (Fisher Scientific) at 50 ppm and incubated for 1 min. After this time, solutions were removed, incubated for 5 min with a Dey/Engley (D/E) neutralizing broth (Becton Dickinson, Sparks, MD, United States) to neutralize the residual chlorine solution, and replaced with BPW. After that, biofilms were scraped from the wells with a pipette tip (three times for periods of 1 min each) and resuspended in BPW (Borges et al., 2017). Biofilm viable counts were measured by standard dilution, plating on Oxford-agar (Oxoid, Lenexa, KS, United States) and MRS-agar, and incubating for 24 and 48 h at 34 and 30°C under aerobic and anaerobic conditions, respectively. The impact of chlorine on cell viability was expressed as the logarithmic value of a relative survivor fraction (log *N* No^–1^), where N refers to the bacterial count following treatments and No refers to the bacterial count avoiding chlorine exposure. The experiment included three replicate wells and was repeated at least three times using independent bacterial cultures.

### Biofilm Growth in Chamber Slides and Fluorescent Labeling

As described above, the inoculum of the best biofilm producers under mono and binary culture conditions was added to 8-well chamber slides (Nunc^TM^ II; Lab-Tek^TM^; Fisher Scientific) at 400 μL per well and let to attach at 34°C for 3 h and then to form biofilms at 34°C for 24 h under aerobic conditions. Each experiment included two replicate chambers and was repeated three times using independent bacterial cultures. After this, the chambers were rinsed with NaCl (8.5 g L^–1^) and refilled with NaCl containing 5 μM Syto^®^9 (1:1,000 dilution from a Syto^®^9 stock solution of the LIVE/DEAD BacLight^TM^ viability kit, Molecular Probes, LifeTechnologies, Eugene, OR), a cell-permeant green fluorescent DNA label. The slide was then incubated in the dark at room temperature for 30 min to enable the fluorescent labeling of the bacteria. Separately (using different chambers), we performed double staining with FITC-WGA (for exopolysaccharide) and SYPRO^®^ Ruby (for proteins). First, FITC-WGA (Sigma-Aldrich) (10 μg mL^–1^) was applied and incubated in the dark at room temperature for 1.5 h. The solution was then removed and the chambers were washed three times with PBS (0.1 M; Sigma-Aldrich). After that, the undiluted FilmTracer^TM^ SYPRO^®^ Ruby Biofilm Matrix Stain (Invitrogen, Carlsbad, CA, United States) was applied and incubated in the dark at room temperature for 30 min. The solution was then removed and the chambers were washed three times with PBS (200 μl each time).

### Confocal Laser Scanning Microscopy (CLSM)

Prior to image acquisition, the plastic chambers were removed from the glass slides and the NaCl solution was applied to the biofilms separated from each other with a gasket. Coverslips were then placed on the gaskets and BacLight^TM^ mounting oil (Molecular Probes) was used to seal their corners, and finally, nail polish was applied to seal the slides. The slides were left overnight at 4°C and observed the day after. Images were acquired with a Zeiss LSM 700 confocal laser scanning microscope (Carl Zeiss Microscopy, Thornwood, NY, United States). All biofilms were scanned using a water-immersion objective lens (Zeiss, 40 × C Pan-Apochromat, NA 1.3) with a 488-nm argon laser and a 561-nm diode-pumped solid-state laser. The fluorescence was recorded within the range from 500 to 600 nm to collect green fluorescence and from 610 to 710 nm to collect red fluorescence. Up to ten stacks of horizontal plane images (260 × 260 μm) with a *z*-step of 0.4 μm were acquired for each chamber in its different areas. Serial images were captured and processed by the Zeiss Zen 2.3 software (Carl Zeiss).

### Image Analysis

Quantitative structural parameters (biomass, biovolume, maximum thickness, average thickness, roughness coefficient, surface to volume ratio (SVR), number of colonies at the substratum, and average colony size at the substratum) were extracted from confocal image series with COMSTAT 2, an image analysis software^1^ (Heydorn et al., 2000; Vorregaard, 2008). Following preliminary analyses, to describe the biofilms under study, we proceeded with the SVR, roughness, and maximum thickness for biofilm architecture and biomass for matrix localization studies.

### Statistical Analysis

All statistical analyses [Principal Component Analysis (PCA), analysis of variance—one-way ANOVA, followed by HSD *post hoc* test] were performed using the Statistica software ver. 13.1 (StatSoft Inc., Tulsa, OK). Differences were considered significant at a *p* < 0.05 level of probability.

## Data Availability Statement

The original contributions presented in the study are included in the article/Supplementary Material, further inquiries can be directed to the corresponding author.

## Author Contributions

MO designed the study, performed the experimental work, analyzed the data, and drafted the manuscript. FD-G revised the manuscript. Both authors contributed to the interpretation of the results, reviewed, and approved the final version of the manuscript.

## Conflict of Interest

The authors declare that the research was conducted in the absence of any commercial or financial relationships that could be construed as a potential conflict of interest.

## Footnotes

1 www.comstat.dk
