# Supplementary material for: Characterization of Binary Biofilms of Listeria monocytogenes and Lactobacillus and Their Response to Chlorine Treatment
Source: Front Microbiol. 2021 Jul 14;12:638933. doi: 10.3389/fmicb.2021.638933 (PMC8317434; doi:10.3389/fmicb.2021.638933)
Supplement: Supplementary file 1 [file Data_Sheet_1.docx]

**TABLE S1** Biofilm formation by *L. monocytogenes* strains.

| Strain | Aerobic conditions | Anaerobic conditions |
| --- | --- | --- |
| F8027 | weak | weak |
| 19115 | strong | moderate |
| ScottA | moderate | weak |
| Jalisco | moderate | weak |
| Bilmar | weak | weak |
| G1091 | moderate | moderate |
| 12443 | weak | weak |
| 51774 | weak | weak |
| FSLJ1-101 | non-adherent | non-adherent |
| F8385 | moderate | moderate |
| 2011L-2626 | weak | weak |
| 51782 | moderate | weak |
| 51779 | weak | weak |
| 108M | weak | non-adherent |
| F6900 | weak | weak |
| Coleslaw | strong | moderate |
| 19117 | strong | moderate |
| F8369 | moderate | weak |
| F8385 | moderate | weak |
| G3982 | moderate | weak |
| 101M | weak | weak |
| G3990 | weak | weak |
| 19114 | moderate | weak |
| G6006 | moderate | weak |
| 51780 | moderate | weak |
| 19116 | moderate | weak |
| F8255 | moderate | weak |

**TABLE S2** Biofilm formation by *Lactobacillus* species.

| **Species** | **Strain** | Aerobic conditions | Anaerobic conditions |
| --- | --- | --- | --- |
| *L. fermentum* | 14931 | strong | moderate |
|  | 36 | weak | non-adherent |
| *L. bavaricus* |  | moderate | weak |
| *L. plantarum* | 2234 | weak | weak |
|  | 17-5 | weak | weak |
|  | CaTC2 | moderate | moderate |
| *L. coprophilus* | 2233 | weak | non-adherent |
| *L. buchneri* | NCDO110 | weak | non-adherent |
| *L. malefermentans* | NCIB8516 | weak | weak |
| *L. sakei* |  | weak | non-adherent |

**TABLE S3** The factor loadings after the varimax rotation of biofilm parameters: SVR, roughness, and maximum thickness for *L. monocytogenes* strains and their nine combination based the PCA.

| Variable | PC 1 | PC 2 |
| --- | --- | --- |
| Max thickness | 0,756081 | -0,563493 |
| Roughness | -0,901053 | 0,021546 |
| SVR | 0,580226 | 0,778279 |
| Lm5 | -0,246549 | 0,123125 |
| Lm7 | -0,068002 | -0,127984 |
| LmC | 0,167762 | -0,277196 |
| Lm5-Lf | 0,044289 | 0,082488 |
| Lm5-Lb | 0,214907 | 0,016790 |
| Lm5-Lp | 0,283284 | 0,592037 |
| Lm7-Lf | 0,151502 | 0,517592 |
| Lm7-Lb | -0,803840 | 0,032044 |
| Lm7-Lp | -0,084115 | -0,215509 |
| LmC-Lf | 0,282887 | -0,528730 |
| LmC-Lb | 0,264634 | -0,166600 |
| LmC-Lp | -0,206760 | -0,048059 |


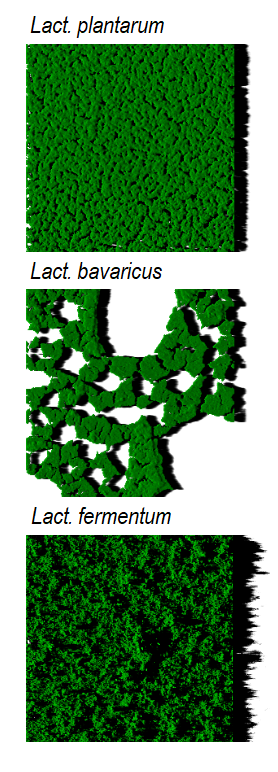


**FIG S1** Three-dimensional biofilm structures of *Lactobacillus* species (*L. plantarum*, *L. bavaricus*, and *L. fermentum*) obtained from confocal z-stacks using ZEN 2.3 software. These images present the shadow projection on the right. The biofilm was labelled with Syto^®^ 9, a cell permeant green fluorescent nucleic acid marker.


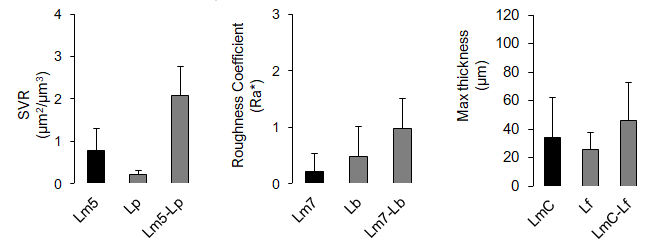


**FIG S2** The structural parameters extracted directly from confocal image stacks for three combinations (Lm5-Lp, Lm7-Lb, and LmC-Lf), including their individual contributions.

**TABLE S4** The factor loadings after the varimax rotation of biomass matrix components for *L. monocytogenes* strains and their nine combination based the PCA.

| **Variable** | **PC 1** | **PC 2** |
| --- | --- | --- |
| Biomass polysaccharides | 0,927926 | 0,375327 |
| Biomass proteins | 0,923609 | -0,380514 |
| Lm5 | -0,251272 | -0,286409 |
| Lm7 | -0,131722 | 0,213338 |
| LmC | -0,392040 | -0,091678 |
| Lm5-Lf | -0,170932 | 0,155013 |
| Lm5-Lb | 0,332521 | 0,031900 |
| Lm5-Lp | 0,328998 | -0,606575 |
| Lm7-Lf | -0,197309 | 0,204822 |
| Lm7-Lb | 0,072025 | -0,443449 |
| Lm7-Lp | 0,570274 | 0,168280 |
| LmC-Lf | 0,280921 | 0,336460 |
| LmC-Lb | -0,043231 | 0,415321 |
| LmC-Lp | -0,398235 | -0,097023 |
